# Conditioned Medium of Bone Marrow Mesenchymal Stem Cells Involved in Acute Lung Injury by Regulating Epithelial Sodium Channels *via* miR-34c

**DOI:** 10.3389/fbioe.2021.640116

**Published:** 2021-07-01

**Authors:** Zhiyu Zhou, Yu Hua, Yan Ding, Yapeng Hou, Tong Yu, Yong Cui, Hongguang Nie

**Affiliations:** ^1^Department of Stem Cells and Regenerative Medicine, College of Basic Medical Science, China Medical University, Shenyang, China; ^2^Department of Anesthesiology, The First Affiliated Hospital of China Medical University, Shenyang, China

**Keywords:** mesenchymal stem cells, conditioned medium, acute lung injury, epithelial sodium channels, miR-34c

## Abstract

**Background:**

One of the characteristics of acute lung injury (ALI) is severe pulmonary edema, which is closely related to alveolar fluid clearance (AFC). Mesenchymal stem cells (MSCs) secrete a wide range of cytokines, growth factors, and microRNA (miRNAs) through paracrine action to participate in the mechanism of pulmonary inflammatory response, which increase the clearance of edema fluid and promote the repair process of ALI. The epithelial sodium channel (ENaC) is the rate-limiting step in the sodium–water transport and edema clearance in the alveolar cavity; the role of bone marrow-derived MSC-conditioned medium (BMSC-CM) in edema clearance and how miRNAs affect ENaC are still seldom known.

**Methods:**

CCK-8 cell proliferation assay was used to detect the effect of BMSC-CM on the survival of alveolar type 2 epithelial (AT2) cells. Real-time polymerase chain reaction (RT-PCR) and western blot were used to detect the expression of ENaC in AT2 cells. The effects of miR-34c on lung fluid absorption were observed in LPS-treated mice *in vivo*, and the transepithelial short-circuit currents in the monolayer of H441 cells were examined by the Ussing chamber setup. Dual luciferase reporter gene assay was used to detect the target gene of miR-34c.

**Results:**

BMSC-CM could increase the viability of mouse AT2 cells. RT-PCR and western blot results showed that BMSC-CM significantly increased the expression of the γ-ENaC subunit in mouse AT2 cells. MiR-34c could restore the AFC and lung wet/dry weight ratio in the ALI animal model, and Ussing chamber assay revealed that miR-34c enhanced the amiloride-sensitive currents associated with ENaC activity in intact H441 cell monolayers. In addition, we observed a higher expression of miR-34c in mouse AT2 cells administrated with BMSC-CM, and the overexpression or inhibition of miR-34c could regulate the expression of ENaC protein and alter the function of ENaC. Finally, we detected that myristoylated alanine-rich C kinase substrate (MARCKS) may be one of the target genes of miR-34c.

**Conclusion:**

Our results indicate that BMSC-CM may alleviate LPS-induced ALI through miR-34c targeting MARCKS and regulate ENaC indirectly, which further explores the benefit of paracrine effects of bone marrow-derived MSCs on edematous ALI.

## Introduction

Acute lung injury (ALI), a common clinical complication, is caused by various intrapulmonary and extrapulmonary factors other than cardiogenic. Alveolar fluid clearance (AFC) is closely related to pulmonary edema, which is one of the characteristics of ALI (Zhang et al., 2017; Han et al., 2020). When AFC is enhanced, the removal of alveolar accumulation fluid is accelerated, which contributes to the relief of pulmonary edema (Deng et al., 2016). In the lung, the epithelial sodium channel (ENaC) is the rate-limiting step in the AFC process, and the primary way to complete the sodium–water transport in alveolar type 2 epithelial (AT2) cells and edema clearance in the alveolar cavity (Ji et al., 2015; Nie et al., 2016; He et al., 2019). A decreased expression of ENaC will lead to the formation of edema, emphasizing that regulation of ENaC is essential for pulmonary edema clearance in patients with edematous ALI (Kellenberger and Schild, 2015).

Mesenchymal stem cells (MSCs) are non-hematopoietic adult stem cells with strong self-renewal ability while maintaining their multipotency (Lee et al., 2009; Walter et al., 2014). They can secrete exosomes and differentiate into a variety of cells under specific induction conditions *in vivo* or *in vitro*, thus helpful in repairing damaged and diseased multiple tissues and organs (Lee et al., 2010; Ornellas et al., 2011). Moreover, they have advantages including easy isolation and better reproductive ability *in vitro* and possessing the characteristics of low immunogenicity (Sakata et al., 2011; Williams and Hare, 2011). In addition, their application does not involve ethical problems (Williams and Hare, 2011; Lee et al., 2013). Therefore, MSCs are widely used in the treatment of various lung diseases in clinical practice, such as ALI, chronic obstructive pulmonary disease, asthma, and pulmonary fibrosis.

MicroRNAs (miRNAs) are small non-coding RNAs, which belong to single-stranded RNA segments with approximately 20–24 nt in length, and are involved in the transcriptional and posttranscriptional modification of protein-coding gene expression (Chen et al., 2017). MSCs can release multiple miRNAs into a conditioned medium (CM), which may act on the receptor cells and are involved in the benefits of re-alveolarization during MSC-mediated reparative processes of an injured respiratory epithelium (Qin et al., 2016). The levels of miRNA expression change according to cell stressors, which may play a critical role in the progression and maintenance of lung diseases (Sessa and Hata, 2013). The translation process of the mRNA molecule is inhibited by complete or incomplete pairing with the complementary sequences of targeting 3′-UTR. Evidence has proved that miRNAs negatively alter the synthesis of the corresponding proteins and ultimately regulate multiple cellular activities (Guo et al., 2010; Banerjee et al., 2020).

Direct administration of MSCs has been shown to be effective in clinical trials, which is definitively the case here, but if the beneficial effects of the MSC-conditioned medium (MSC-CM) are as good as those seen with cell-based therapy beyond the contact-dependent mechanisms of MSCs, this therapeutic strategy would be simpler and with fewer potential limitations (Hwang et al., 2016). Lots of studies have reported the effects of bone marrow-derived MSCs (BMSCs) on ALI, but it has rarely been reported that bone marrow-derived MSC-conditioned medium (BMSC-CM) reliefs edematous ALI by regulating alveolar epithelial ion transport, and AFC would be quite indicative of this metric. In this paper, we researched BMSC-CM in exploring cell-free therapy and identified that miRNAs released by BMSC-CM could regulate ENaC to improve edema clearance, which may be considered as the interesting target for treating edematous lung disease development and providing a new pharmacological strategy as well.

## Materials and Methods

### Animals

Pathogen-free C57 mice were purchased from Liaoning Changsheng Biotechnology Co., Ltd., and the animal certificate number is SCXK (Liao) 2018-0001. All experimental methods involving C57 mice were performed in accordance with the guidelines of the Animal Care and Use Ethics Committee (Certificate Number: CMU2019088), and all protocols were agreed with China Medical University. Mice were kept without pathogens and kept with free water and chow access. All animals were housed in an air-conditioned room on a 12-h light/12-h dark cycle.

### Isolation, Culture, and Identification of Mouse BMSCs and AT2 Cells

Three-week-old pathogen-free male C57 mice weighing 9–13 g were anaesthetised by diazepam (17.5 mg kg^–1^) followed 6 min later by ketamine (450 mg kg^–1^) intraperitoneally. We isolated the femora and collected the bone marrow by washing the femora medullary cavity with DMEM/F12 medium (HyClone) plus 10% fetal bovine serum (FBS, Gibco), 10 ng/ml bFGF (PeproTech, Rocky Hill, NJ, United States), and antibiotics. At the end of the study, all mice were anesthetized by being transferred to a facemask carrying 2.5% isoflurane, euthanized by cervical dislocation. BMSCs were cultured in the condition of 5% CO_2_, 37°C for 24 h, and the medium was replaced every other day. At 80% confluence, the medium of BMSCs was changed with DMEM/F12 medium without FBS. BMSC-CM was collected after 24 h and stored at −80°C.

Lung stripping of newborn C57 mice (within 24 h) was placed in prechilled PBS and then minced, digested with 0.25% trypsin and 0.1% type I collagenase at 37°C for 30 min, respectively. We filtered and cultured cells in 5% CO_2_, 37°C, in DMEM/F12 plus 10% FBS, and antibiotics for 45 min. Unattached cells were collected, and we repeated the above culture steps for four times to remove the fibroblast cells. Next, the cell suspension was delivered to an IgG-coated culture dish and incubated for 30 min. We adjusted the unattached cells to 2–3 × 10^6^/ml and changed the medium 72 h later, then replaced them every 48 h.

### CCK-8 Cell Proliferation Assay

Primary mouse AT2 cells were seeded in 24-well plates (∼4 × 10^5^ cells/well) and cultured them in DMEM/F12 medium containing 10% FBS in a 5% CO_2_, 37°C cell incubator for 12 h. Thereafter, the medium containing 10% CCK-8 was added and the cells were incubated for 1 h in the dark. The initial OD (0 point) value was measured at 450 nm using a microplate reader. The cells in the 24-well plate were randomly divided into four groups: the first group was cultured in normal DMEM/F12 medium containing 10% FBS (control group, Control), the second group was cultured in serum-free DMEM/F12 medium (serum-free group, SD), the third group was cocultured with BMSCs (coculture group, Co-culture), and the fourth group was administrated with BMSC-CM (CM group). After 24 h, the original medium was aspirated, and the DMEM/F12 medium containing 10% CCK-8 was added to measure the OD value and calculate the cell survival rate accordingly.

### Ussing Chamber Assay

NCI-H441 cells were purchased from ATCC and cultured as previously described (Ding et al., 2020). Briefly, H441 cells were grown in RPMI medium (ATCC) with 10% FBS, and for Ussing chamber recording, cells were planted on permeable Transwells (Costar) at a density nearly 5 × 10^6^ cells/cm^2^ and incubated in a condition of 5% CO_2_, 37°C. After seeding, cells reached confluence in the Costar support filters after 24 h. Then we removed the medium in the apical compartment to expose the cells to air–liquid interface growth. The culture medium of the basolateral compartment was replaced every 48 h; meanwhile, the apical medium was changed with PBS. Transepithelial resistance was recorded with a volt–ohm meter of epithelial tissue (World Precision Instruments, Sarasota, FL, United States). When the resistance is higher than 500 Ω cm^2^, the polarized tight monolayers could be used for measuring transepithelial short-circuit current (Isc).

Measurements of transepithelial Isc and resistance were done as previously described (Nie et al., 2016). Briefly, we mounted H441 monolayers in Ussing chambers (Physiologic Instruments, San Diego, CA, United States), which were bathed with a solution on both sides including the following (in mM): 120 NaCl, 25 NaHCO_3_, 3.3 KH_2_PO_4_, 0.83 K_2_HPO_4_, 1.2 CaCl_2_, 1.2 MgCl_2_, 10 HEPES sodium salt, and 10 mannitol/D-glucose for apical/basolateral compartment. The pH for each solution was adjusted to 7.4, and the solutions of both sides were bubbled with mixed gas at 37°C. We measured Isc with Ag–AgCl electrodes prefilled with 4% agar dissolved with 3 M KCl. The monolayers were short-circuited to 0 mV, and a 10-mV pulse of 1 s was applied every 10 s to monitor the transepithelium resistance. The Acquire and Analyze 2.3 program was used to collect data, and ENaC activity was the decreased level of Isc after the administration of 100 μM amiloride to the apical side.

### Western Blot Assay

H441 and mouse AT2 cells were cultured in six-well plates, washed with PBS when cells were fused to 80%, and then assayed by immunoblotting. Proteins were separated on 10% SDS-PAGE gels and transferred to PVDF membranes (Invitrogen). The blots were immersed in blocking solution with 20 mM Tris–HCl (pH 7.5), 1% Tween (TBST), 0.5 M NaCl, and 5% BSA for 1 h. Membranes were incubated with 5% BSA of γ-ENaC antibody (Thermo Fisher, Waltham, MA, United States) or β-actin (Santa Cruz Biotechnology, Santa Cruz, CA, United States) overnight at 4°C. Following washing three times with TBST, membranes were incubated with HRP-conjugated goat-anti-rabbit/goat-anti-mouse secondary antibody for 1 h at room temperature, and then washed three times with TBST for 10 min. Images were developed using an enhanced ECL kit and analyzed with the ImageJ program.

### Real-Time Polymerase Chain Reaction

Total RNA was extracted with TRIzol reagent (Invitrogen) according to the manufacturer’s instructions. We used spectrophotometry to measure the concentration/purity of total RNA. Reverse transcription was performed with a PrimeScript RT Reagent Kit and gDNA Eraser (TaKaRa). Real-time polymerase chain reaction (RT-PCR) with SYBR Premix Ex Taq II (TaKaRa, Kusatsu, Japan) was performed using ABI7500. GAPDH was used as a reference. The following primer pairs were used: γ-ENaC, 5′-GCACCG TTC GCC ACC TTC TA-3′ (sense), 5′-AGG TCA CCA GCA GCT CCT CA-3′ (antisense); GAPDH, 5′-AGA AGG CTG GGG CTC ATT TG-3′ (sense), 5′-AGG GGC CAT CCA CAG TCT TC-3′ (antisense); and miR-34c, 5′-AGG CAG UGU AGU UAG CUG AUU GC-3′ (sense), 5′-AAU CAG CUA ACU ACA CUG CCU UU-3′ (antisense). The expression levels of miR-34c were measured using the Mir-X miRNA First-Strand Synthesis Kit (TaKaRa). U6 was used as a reference, and the final volume is 15 μl. The reaction conditions of the miRNA were a single cycle of 95°C for 30 s, 5 s at 95°C, and 40 cycles at 60°C for 30 s. The data from RT-PCR were analyzed using the 2^–△△^^*CT*^ method.

### AFC Measurement and Lung Wet/Dry Weight Ratio in LPS-Treated ALI Mice

LPS was administrated to set up the ALI model in mice, which was one of clinical symptoms during the endotoxic shock induced by LPS. Mice were randomly divided into Control, LPS, and LPS + miR-34c groups. MiR-34c was injected into mice through the caudal vein (2 mg/kg) every 24 h for three successive days, then mice were administrated with LPS (5 mg/kg) intraperitoneally. Mice were injected with equal volumes of normal saline in the Control group. After another 24 h, AFC was measured and the lung tissues were extracted for lung wet/dry weight (W/D) ratio calculation. AFC was performed *in vivo* as previously mentioned (Nie et al., 2016). Briefly, C57 mice were supplied with 100% O_2_ *via* a ventilator (Chengdu Taimeng Co. Ltd., Chengdu, China) after being anesthetized with 17.5 mg/kg diazepam and 450 mg/kg ketamine intraperitoneally. Five percent bovine serum albumin (BSA) (200 μl) solution was instilled intratracheally, and the alveolar fluid was collected 30 min later. AFC was calculated as the following formula: AFC = [(Vi − Vf)/Vi] × 100, in which Vi and Vf indicate the volume of the alveolar fluid instilled and collected, respectively. Vf = (Vi × Pi)/Pf, where Pi and Pf represent the BSA concentration in the instilled and collected fluid, respectively. The lung tissues were weighed and then placed in an incubator at 80°C for 48 h to obtain the dry weight. The W/D ratio was calculated as the ratio of lung wet weight and dry weight.

### Transfections

H441 and AT2 cells were cultured in a six-well plate. When the cells were fused to 50–60%, the serum-containing medium was discarded, and then the cells were replaced with serum-free medium after washing with PBS. Negative control (NC), miR-34c mimics, inhibitor NC, or miR-34c inhibitor (GenePharma, Shanghai, China) was transfected into cells with siRNA-mate according to the manufacturer’s instructions. The final concentration of miR-34c mimics was 30 nM, and the miR-34c inhibitor was 60 nM. All transfection reagents were discarded after 6 h, and cells were used 48 h posttransfection.

### Dual Luciferase Reporter Gene Assay

The dual luciferase reporter gene detects the regulation of genes by reflecting the amount of luciferase expression. H441 cells were cultured in a six-well plate and fused to 60–70%. The serum-containing medium was discarded, and the cells were replaced with serum-free medium. The constructed myristoylated alanine-rich C kinase substrate (MARCKS)-3′UTR wild-type (WT) and mutant recombinant plasmids (GenePharma) were transfected into H441 cells with miR-34c mimics or NC, respectively. After 48 h, luciferase activity was measured using the Dual Luciferase Reporter Assay Kit (Vazyme, Nanjing, China), according to the manufacturer’s instructions.

### Statistical Analysis

Data were expressed as the mean ± SE. We evaluated the power of sample size first to meet *P* < 0.05, then examined whether the data were parametric or not. Normality and homoscedasticity tests were done by Levene and Shapiro–Wilk tests before applying parametric tests. For comparing two groups, we applied Student’s two-tailed *t*-test; for comparing multiple groups, we applied one-way analysis of variance (ANOVA) followed by Bonferroni test for all the groups of the experiment. When the data did not conform to the normality or homoscedasticity test, we performed a non-parametric *t*-test (Mann–Whitney *U*-test). We considered variations significant when the *P*-value was < 0.05. Statistical analysis was done with Origin 8.0.

## Results

### BMSCs and BMSC-CM Enhance the Viability of Mouse AT2 and H441 Cells

**FIGURE 1:**
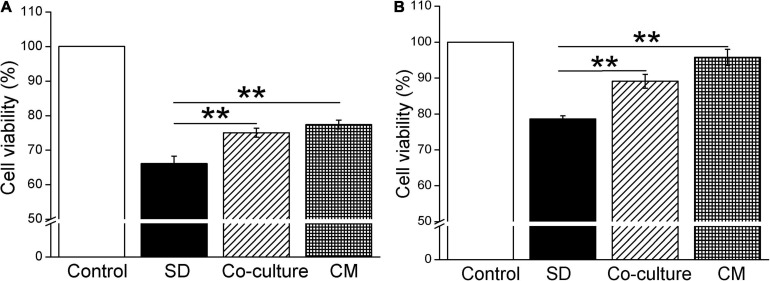
Bone marrow-derived MSCs enhance the cell viability in mouse AT2 and H441 cells. CCK-8 cell proliferation assay under the condition of normal medium (Control), serum-free medium (SD), coculture with BMSCs (co-culture), and BMSC-CM administration in AT2 **(A)** and H441 **(B)** cells. **P < 0.01, compared with the SD group, *n* = 5 ∼ 6.

To investigate the effect of BMSCs on the cell viability by CCK-8 cell proliferation assay, we first cocultured BMSCs with primary mouse AT2 and H441 cells, respectively. As shown in Figure 1A, coculture of BMSCs and primary AT2 cells significantly improved cell survival, compared with the serum-free (SD) group (*P* < 0.01, *n* = 6). Meanwhile, administration of BMSC-CM also increased the viability of AT2 cells (*P* < 0.01, *n* = 6, compared with the SD group). As expected, BMSCs and BMSC-CM were also able to increase the survival rate in H441 cells (*P* < 0.01, *n* = 5, compared with the SD group, Figure 1B). By applying Trypan Blue Counterstain and counting the number of live/dead staining cells, respectively, we found that both the numbers of total and live cells increased, supporting that both BMSCs and MSC-CM could enhance the viability of the cells by promoting the proliferation of cells (Supplementary Figure 1), and we administrated BMSC-CM for the cell treatment in the following experiments.

### BMSC-CM Increases the Protein and mRNA Expression of γ-ENaC in Mouse AT2 and H441 Cells

**FIGURE 2:**
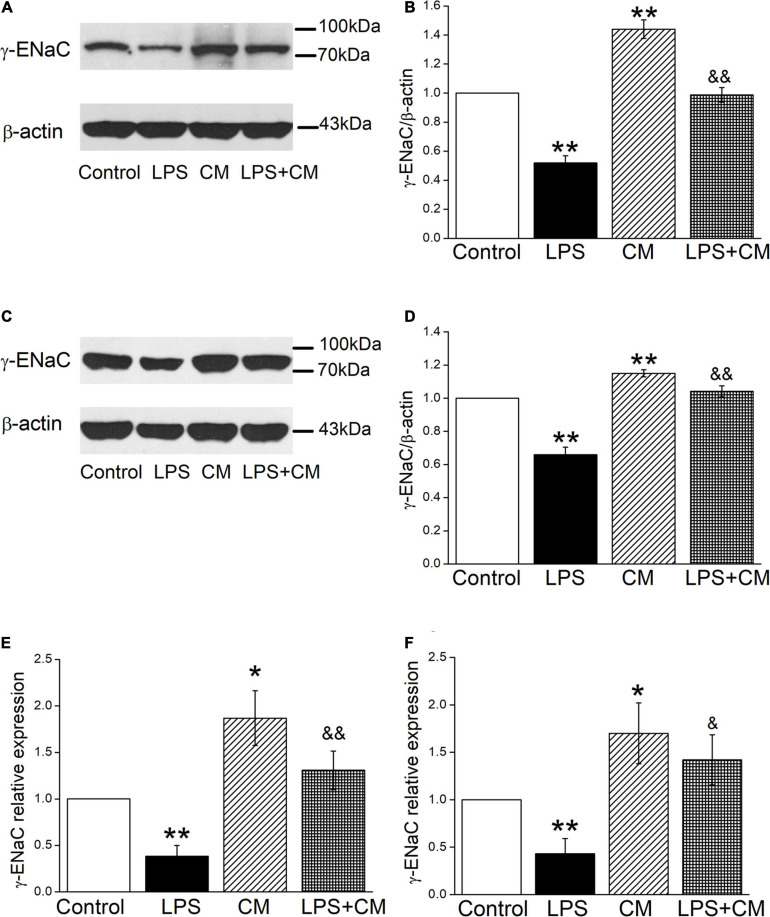
Bone marrow-derived MSC-conditioned medium increases the protein and mRNA expression of γ-ENaC after LPS administration. **(A,C)** Representative cropping gel blots of γ-ENaC protein in control (Control), LPS (10 μg/ml, 12 h, LPS), BMSC-CM (24 h, CM), and BMSC-CM plus LPS (LPS + CM) group of mouse AT2 and H441 cells, respectively. **(B,D)** Graphical representation of data obtained from western blots and quantified through gray analysis (γ-ENaC/β-actin). **(E,F)** Real-time PCR results of γ-ENaC mRNA expression in mouse AT2 cells and H441 cells, respectively. *P < 0.05, **P < 0.01, compared with the Control group. ^&^*P* < 0.05, ^&&^*P* < 0.01, compared with the LPS group. *n* = 5 ∼ 7.

We further examined the effects of BMSC-CM on ENaC at the protein expression levels in mouse AT2 and H441 cells, respectively. As shown in Figures 2A,B, a specific band of γ-ENaC between 70 and 100 kD was observed by western blot assay according to the manufacturer’s manual. LPS downregulated γ-ENaC expression in AT2 cells compared with the Control group (*P* < 0.01), and BMSC-CM increased the protein expression of γ-ENaC in both primary and LPS-treated AT2 cells (*P* < 0.01 compared with the Control and LPS groups, respectively, *n* = 5). Similar results were observed in H441 cells and the LPS-induced ALI cell model (Figures 2C,D, *P* < 0.01, compared with the Control and LPS groups, respectively, *n* = 7). We speculate that the higher protein expression of γ-ENaC was associated with the increased transcription level, which was confirmed by the RT-PCR results that administration of BMSC-CM caused a significant increase of γ-ENaC mRNA expression in LPS-treated primary AT2 and H441 cells (*P* < 0.01 and *P* < 0.05 compared with the LPS group, respectively, *n* = 5, Figures 2E,F).

### BMSC-CM Enhances the Expression of miR-34 in Mouse AT2 Cells

**FIGURE 3:**
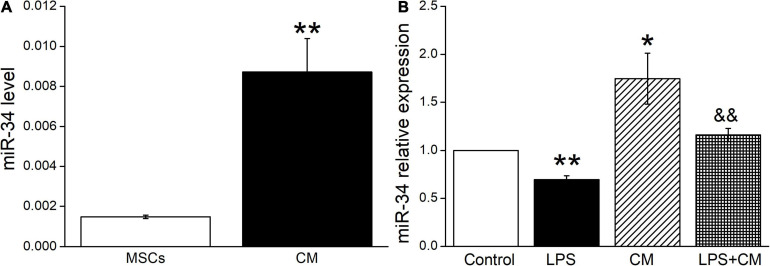
MiR-34c is present in BMSC-CM, and the expression of miR-34c is decreased in LPS-treated mouse AT2 cells. **(A)** The MiR-34c level in BMSCs (MSCs) and BMSC-CM (CM). **P < 0.01, compared with the MSC group. **(B)** The result of real-time PCR assay showing the miR-34c level in control AT2 cells (Control), LPS (10 μg/ml, 12 h, LPS), BMSC-CM (24 h, CM), and BMSC-CM plus LPS (LPS + CM) groups of mouse AT2 cells. The relative level of miR-34c was calculated as miR-34c/U6 ratios. *P < 0.05, **P < 0.01, compared with the Control group. ^&&^*P* < 0.01, compared with the LPS group. *n* = 6.

Previous studies suggested that miR-34c was involved in the corresponding pathways related to LPS-induced ALI (Zhu et al., 2019). In our experiment, we first measured the miR-34c level in BMSCs and BMSC-CM, respectively, and the data showed a higher expression in BMSC-CM (Figure 3A). Next, we analyzed the expression levels of miR-34c in mouse AT2 cells after BMSC-CM administration. As shown in Figure 3B, we found that exposure to LPS caused a significant decrease in miR-34c level compared with the Control group (*P* < 0.01). Conversely, the levels of miR-34 increased in normal and LPS-treated mouse AT2 cells after administration of BMSC-CM (*P* < 0.05 and *P* < 0.01 compared with the Control and LPS groups, *n* = 6), respectively. These data proved that the expression level of miR-34c decreased during ALI and BMSC-CM upregulated the level of miR-34c in the LPS-induced ALI cell model.

### MiR-34c Restores AFC and Decreases Lung W/D Ratio in LPS-Treated Mice

**FIGURE 4:**
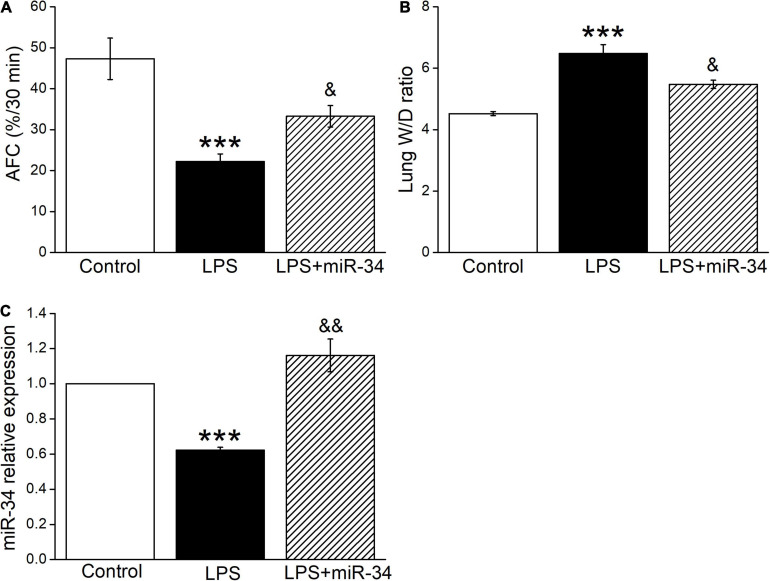
MiR-34c restores AFC and decreases the lung wet/dry weight ratio in LPS-treated mice. **(A)** Mouse lungs were treated with 5% bovine serum albumin dissolved in physiologic saline solution in the vehicle-treated control group (Control), LPS group (5 mg/kg, 24 h, LPS), and LPS plus miR-34c mimics group (2 mg/kg, 72 h, LPS + miR-34), respectively. The reabsorption rate of instillate was computed as the percentage of instilled volume after 30 min (AFC%/30 min). **(B)** The lung wet/dry weight (W/D) ratio. **(C)** MiR-34c relative expression level by real-time PCR assay. ***P < 0.001, compared with the Control group. ^&^*P* < 0.05, ^&&^*P* < 0.01, compared with the LPS group. *n* = 5.

To clarify the function of miR-34c on lung fluid transport *in vivo*, we measured both AFC and W/D ratio in LPS-treated mice, which reflected more directly about the fluid retention in ALI. As expected, AFC was decreased after LPS administration compared with the Control group (*P* < 0.001, Figure 4A), while miR-34c could partially restore the LPS-inhibited AFC (*P* < 0.05, compared with the LPS group). As for the W/D ratio, miR-34c reversed the LPS-enhanced W/D ratio (*P* < 0.05, compared with the LPS group), supporting the evidence that miR-34c could increase lung fluid transport in the LPS-induced ALI animal model (Figure 4B). The miR-34c expression level in mice treated with miR-34c injection was proved by RT-PCR, which is shown in Figure 4C (*P* < 0.01, compared with the LPS group, *n* = 5).

### The Level of miR-34c Is Positively Correlated With γ-ENaC in Mouse AT2 Cells

**FIGURE 5:**
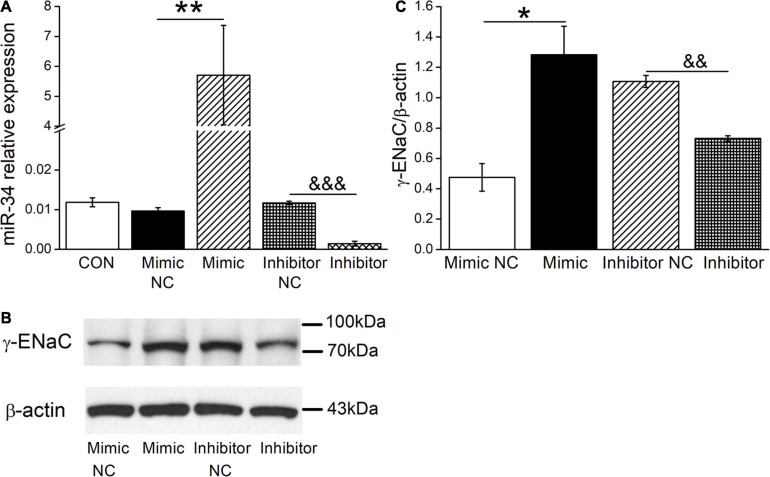
The levels of miR-34c and γ-ENaC are positively correlated in mouse AT2 cells. **(A)** Transfection efficiency of miR-34c in mouse AT2 cells. Cells were transfected with control (CON), negative control (Mimic NC), miR-34c mimics (Mimic), inhibitor negative control (Inhibitor NC), or miR-34c inhibitor (Inhibitor), respectively. **P < 0.01, compared with the Mimic NC group. ^&&&^*P* < 0.001, compared with the inhibitor NC group. *n* = 4. **(B)** Representative cropping gel blots of γ-ENaC protein in AT2 cells transfected with miR-34c negative control (Mimic NC), miR-34c mimics (Mimic), inhibitor negative control (Inhibitor NC), or miR-34c inhibitor (Inhibitor). **(C)** Graphical representation of data obtained from western blots and quantified through gray analysis (γ-ENaC/β-actin). *P < 0.05, compared with the Mimic NC group. ^&&^*P* < 0.01, compared with the Inhibitor NC group. *n* = 3.

Based on the above facts that BMSC-CM could both upregulate the expression levels of γ-ENaC and miR-34c, we assume that BMSC-CM may enhance the expression of γ-ENaC protein through miR-34c accordingly. To test this hypothesis, mouse AT2 cells were transfected with miR-34c mimics (Mimic) or inhibitor (Inhibitor), respectively. The transfection efficiency of miR-34c was verified by RT-PCR (*n* = 4, Figure 5A). The effect of miR-34c on γ-ENaC was examined by Western blot analysis, and the histogram was illustrated for the sake of comparison (Figures 5B,C). Transfection of miR-34c mimics into mouse AT2 cells resulted in a significant increase of γ-ENaC expression compared with the NC (Mimic NC) group (*P* < 0.05), while inhibition of miR-34c showed opposite effects (*P* < 0.01, *n* = 3). These data suggested that miR-34c increased the protein expression of γ-ENaC. The potential mechanism of BMSC-CM protection in ALI may be related to the enhanced miR-34c level and sequent stimulation of γ-ENaC protein expression.

### MiR-34c Increases Transepithelial Short-Circuit Current in Confluent H441 Monolayers

**FIGURE 6:**
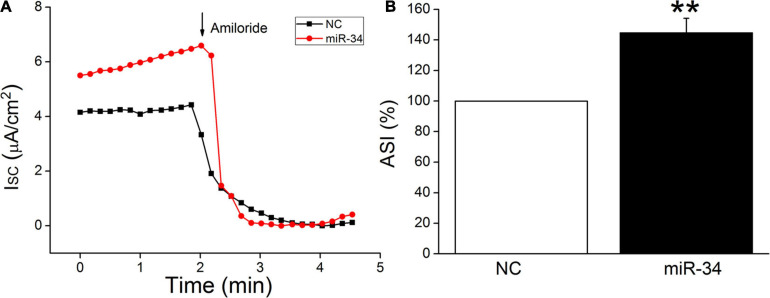
MiR-34c increases amiloride-sensitive short-circuit current in H441 monolayers. **(A)** Representative short-circuit current (Isc) traces recorded in confluent H441 monolayers transfected with miR-34c negative control (NC) and miR-34c mimics (miR-34) for 48 h. **(B)** Percentage of amiloride-sensitive Isc (ASI%) in the NC and miR-34 groups. ASI was defined as the difference between the total current and the amiloride-resistant current, and the negative control (NC) was set as 100%. **P < 0.01, compared with the NC group. *n* = 5.

H441 cells are derived from human lung Clara cells, which can easily form monolayers at the air–liquid interface and exhibit a number of recognized morphological structures and functional similarities to primary human alveolar type II cells (Albert et al., 2008; Korbmacher et al., 2014). This cell line has been widely used to study the function of ENaC in the lung, and the ENaC characteristics of H441 were similar to those of primary AT2 cells, which could hardly grow into monolayers (Demaio et al., 2009; Nie et al., 2009, 2016). In our recent study, BMSC-CM has been proved to activate amiloride-sensitive Isc (ASI), which reflects ENaC activity in LPS-treated H441 monolayers (Ding et al., 2020). In order to further confirm the functional regulation of ENaC by miR-34c, we measured Isc in confluent H441 monolayers. As shown in Figure 6, ASI was defined as the difference between the total current and the amiloride-resistant current, and the NC was set as 100%. MiR-34 significantly increased the ASI of H441 monolayers to 132.3 ± 8.4% (*P* < 0.01, compared with NC, *n* = 5), which indicates that miR-34c may enhance the fluid transport of lung through increasing ENaC activity in alveolar epithelial cells.

### MiR-34c Upregulates ENaC Expression by Binding to the 3′-UTR of MARCKS

**FIGURE 7:**
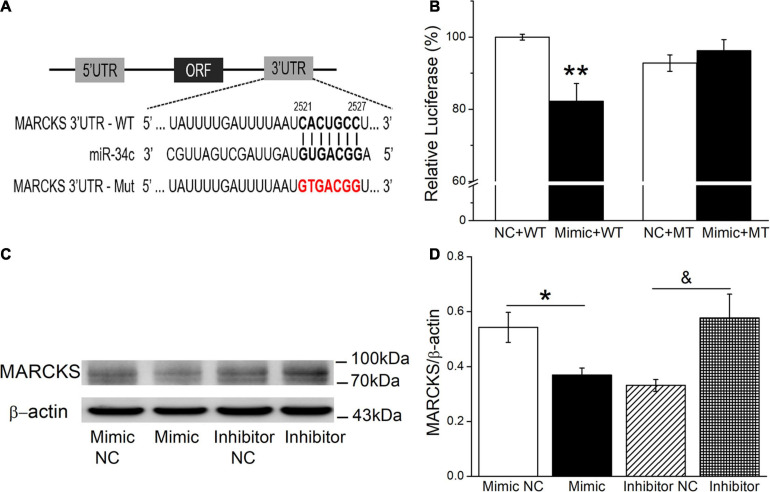
Dual luciferase assay for miR-34c binding with MARCKS in mouse AT2 cells. **(A)** The potential binding sites for miR-34c on the 3′-UTRs of MARCKS. **(B)** AT2 cells were co-transfected with negative control (NC) or miR-34c mimics (Mimic) together with pmirGLO-MARCKS (WT or MT) for 24 h. **P < 0.01, compared with the NC group. *n* = 5. **(C)** Representative cropping gel blots of MARCKS protein in AT2 cells transfected with miR-34c negative control (Mimic NC), miR-34c mimics (Mimic), inhibitor negative control (Inhibitor NC), or miR-34c inhibitor (Inhibitor). **(D)** Graphical representation of data obtained from western blots and quantified through gray analysis (MARCKS/β-actin). *P < 0.05, compared with the Mimic NC group. ^&^*P* < 0.05, compared with the Inhibitor NC group. *n* = 4.

Potential miR-34c targets were predicted according to the bioinformatic websites, including http://www.informatics.jax.org/, http://mirwalk.umm.uni-heidelberg.de/, and http://mirdb.org/index.html (Supplementary Figure 2). In view of MARCKS as a ubiquitous and highly conserved protein, which plays important roles in secretion, transmembrane transport, and regulation of many molecular interactions, especially also as a regulator of ENaC activity, we chose MARCKS as a target for our future investigation (Alli et al., 2015; Montgomery et al., 2017). To confirm this finding, a dual luciferase target gene assay was conducted. WT and mutant reporter gene vectors (MT) for MARCKS were constructed, and as shown in Figure 7A, mouse AT2 cells were co-transfected with the vectors (NC) and miR-34c mimics (Mimic). After 24 h, the *firefly* luciferase activity was measured, then normalized with *Renilla* luciferase units (Figure 7B). We found that the expression of pmirGLO-MARCKS-WT (Mimic+WT) relative luciferase activity was reduced significantly by miR-34c (*P* < 0.01, compared with NC+WT), while the expression of pmirGLO-MARCKS-MT (Mimic+MT) was not suppressed by miR-34c (*P* > 0.05, compared with NC+MT, *n* = 5). Western blot assay also identified that miR-34c mimics evidently inhibited the protein expression of MARCKS, while inhibition of miR-34c showed opposite effects (Figures 7C,D, *P* < 0.05, *n* = 4). The above data verified that MARCKS was one of the target genes of miR-34c, which may upregulate ENaC expression by binding to the 3′-UTR of MARCKS.

## Discussion

Mesenchymal stem cell are being extensively investigated for their potential benefits in tissue engineering and regenerative medicine (Sigmarsdottir et al., 2020). Of note, MSCs have many advantages, such as extensive sources, easy to culture *in vitro*, and less ethical issues, hence the study of MSCs is more extensive at present. Researchers have reported that MSCs can exert beneficial effects by their released extracellular vesicles; moreover, the therapeutic strategy of MSC-CM would be more beneficial than cell-based therapy, in which potential limitations existed. It has been confirmed that patients with ALI have disorders of pulmonary fluid clearance, and evidence has also shown that reabsorption of Na^+^ by ENaC is an important step to maintaining the balance of alveolar fluid (Chang et al., 2018). However, whether ENaC is involved in the above process is still rarely known (Tsiapalis and O’Driscoll, 2020).

In our experiment, we first used CCK-8 cell proliferation assay to prove the influence of BMSC-CM on the viability of alveolar epithelial cells, which could increase the survival rate of both mouse AT2 and H441 cells. In order to verify whether BMSC-CM could regulate ENaC, we next studied the effect of BMSC-CM on ENaC expression in mouse AT2 cells. The stimulation of α-ENaC in AT2 cells has been proved in our recent study; we further identified the similar enhancement of γ-ENaC expression by BMSC-CM at protein and mRNA levels (Ding et al., 2020).

The miRNAs are short (approximately 22 nt) and non-coding RNAs, which function as posttranscriptional repressors of gene expression by binding predominantly to the 3′-UTR of mRNAs to interfere with protein production (Flynt and Lai, 2008; Bartel, 2009). The involvement of miRNAs in ALI is still seldom known (Zhou et al., 2019). We tried TargetScan and Miranda software for the prediction, while using DIANA-microT to screen the identical miRNAs. Based on the upregulation of γ-ENaC and possibly beneficial effects of BMSC-CM in ALI, we speculate that the miRNAs secreted by BMSCs to the medium, especially miR-34c present in BMSC-CM, might be involved with LPS-induced ALI (Zhu et al., 2019). The expression of miR-34c decreased in the LPS group, whereas it increased significantly in the BMSC-CM group, indicating that miR-34c might participate in the ENaC regulation of BMSC-CM in alveolar epithelial cells.

The evidence that BMSC-CM could increase the protein and mRNA expression of ENaC and the expression of miR-34c was higher in BMSC-CM made us suppose that miR-34c might also be involved in the beneficial effect of BMSC-CM in ALI. We measured both AFC and W/D ratio in the LPS-treated ALI mouse model, which reflected more directly about the fluid absorption after miR-34c administration. Then, we transfected miR-34c mimics or inhibitor into mouse AT2 cells, respectively. As expected, western blot analysis showed that miR-34c could significantly increase the protein expression of γ-ENaC, while the inhibitor of miR-34c exhibited the opposite effects. The human lung adenocarcinoma cell line H441 is one of the classical cell lines for studying the function and activity of ENaC. In our previous studies, Ussing chamber assay was used to record the Isc in confluent H441 monolayers (Nie et al., 2016). Besides ENaC, there are many other channels involved in the regulation of Isc, like Na^+^, K^+^-ATPase, cystic fibrosis transmembrane conductance regulator (CFTR), Na^+^-K^+^-2Cl^–^ co-transporter (NKCC), Ca^2+^-activated K^+^ channel (KCa), and Ca^2+^-activated Cl^–^ channel (CaCC). To emphasize the key point of ENaC, which is the rate-limiting step in the sodium–water transport of alveolar epithelial cells, we calculated the ASI which reflected ENaC activity and revealed that miR-34c could increase ASI in intact H441 monolayers.

**FIGURE 8:**
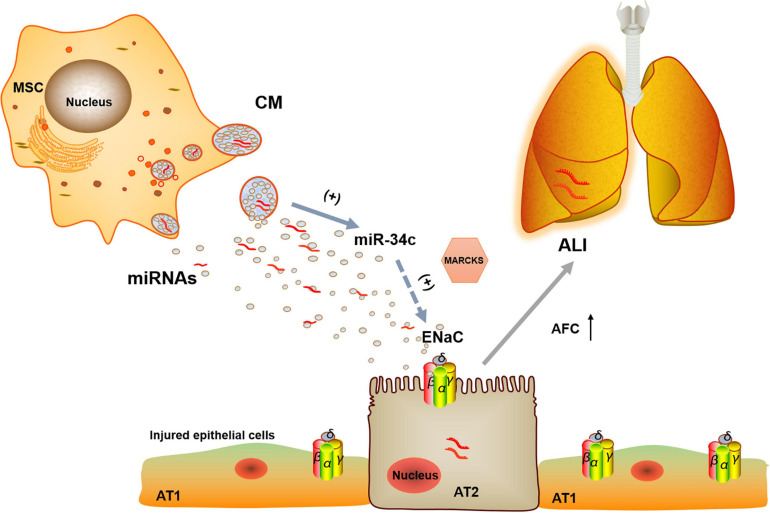
Potential mechanisms for miR-34c involved in MSC-CM regulation of LPS-induced ALI. MiR-34c can bind 3′-UTR of MARKS and increase γ-ENaC expression and function indirectly in alveolar epithelial cells, which may enhance AFC and relieve MSC-CM-regulated edematous ALI. MSC, mesenchymal stem cell; CM, conditioned medium; ENaC, epithelial sodium channel; AFC, alveolar fluid clearance; miRNAs, microRNAs; AT1, alveolar type 1 epithelial cells; AT2, alveolar type 2 epithelial cells; ALI, acute lung injury.

From above, we can see that BMSC-CM could protect edematous lung injury by increasing the protein and mRNA expression of γ-ENaC, which may be related with miR-34c. Then, a question arose: how did miR-34c regulate γ-ENaC? The NC of miRNAs and their target genes almost excluded the direct binding of miR-34c with ENaC, and we investigated the possible regulator MARCKS, which was both an upstream factor of ENaC and a possible effector of miR-34c. The double luciferase reporter gene identified the direct binding of miR-34c with MARCKS, whereas MARCKS phosphorylation was found to decrease ASI in a time- and dose-dependent manner and ENaC activity could also be regulated by calpain-2 proteolysis of MARCKS proteins (Alli et al., 2012; Montgomery et al., 2017). Our results showed that miR-34c could act on γ-ENaC indirectly, and MARCKS might be one of its target genes, through which miR-34c inhibited the expression and activity of γ-ENaC (Figure 8). This topic studied the possible mechanisms of BMSC-CM in the treatment of LPS-induced ALI at the molecular level, which would provide a theoretical basis and therapy target for ALI related edematous lung diseases.

## Conclusion

Bone marrow-derived MSC-conditioned medium can increase LPS-induced ENaC expression and function involved in ALI at least by upregulating miR-34c, and MARCKS is one of its target genes. These findings will further explore the therapeutic effects of paracrine effects of BMSCs on edematous lung diseases.

## Data Availability Statement

The raw data supporting the conclusion of this article will be made available by the authors, without undue reservation.

## Ethics Statement

The animal study was reviewed and approved by Local Ethics Committee for the Care and Use of Laboratory Animals of China Medical University.

## Author Contributions

HN conceived and designed the study. ZZ, YC, YH, and YD performed the study. ZZ and TY analyzed the data. ZZ and HN drafted the manuscript. YC revised the draft of the manuscript. All authors corrected and approved the final version of the manuscript.

## Conflict of Interest

The authors declare that the research was conducted in the absence of any commercial or financial relationships that could be construed as a potential conflict of interest.
