# Supplementary material for: Conditioned Medium of Bone Marrow Mesenchymal Stem Cells Involved in Acute Lung Injury by Regulating Epithelial Sodium Channels via miR-34c
Source: Front Bioeng Biotechnol. 2021 Jul 1;9:640116. doi: 10.3389/fbioe.2021.640116 (PMC8336867; doi:10.3389/fbioe.2021.640116)
Supplement: Supplementary file 1 [file Data_Sheet_1.docx]

**Conditioned Medium of Bone Marrow Mesenchymal Stem Cells Involved in Acute Lung Injury by Regulating Epithelial Sodium Channels *via* miR-34c**

**Zhiyu Zhou^1^, Yu Hua^1^, Yan Ding^1^, Yapeng Hou^1^, Tong Yu^1^, Yong Cui^2^**^*^**, and Hongguang Nie^1*^**

*^1^Department of Stem Cells and Regenerative Medicine, College of Basic Medical Science, China Medical University, Shenyang, 110122, China*

*^2^Department of Anesthesiology, the First Affiliated Hospital of China Medical University, Shenyang, 110001, China*

*Correspondence: Hongguang Nie, [hgnie@cmu.edu.cn](mailto:hgnie@cmu.edu.cn); Yong Cui, cynhg@sina.com

**Figure Legends:**

**SUPPLEMENTARY FIGURE 1.** BMSCs enhances both the cell numbers of total and live cells in mouse AT2 cells. Trypan Blue Counterstain were used to count the number of live/dead staining cells, under the condition of normal medium (Control), serum-free medium (SD), co-culture with BMSCs (Co-culture), and BMSCs-CM administration in AT2 cells. ^*^*P* < 0.05, ^&&^*P* < 0.01, compared with total and live cells in SD group, respectively, n = 6.

**SUPPLEMENTARY FIGURE 2.** Potential miR-34c target of MARKS was predicted according to the bioinformatic websites. (**A**) <http://www.informatics.jax.org/> (**B**) <http://mirwalk.umm.uni-heidelberg.de/> and (**C**) <http://mirdb.org/index.html>.

**SUPPLEMENTARY FIGURE 1**

**
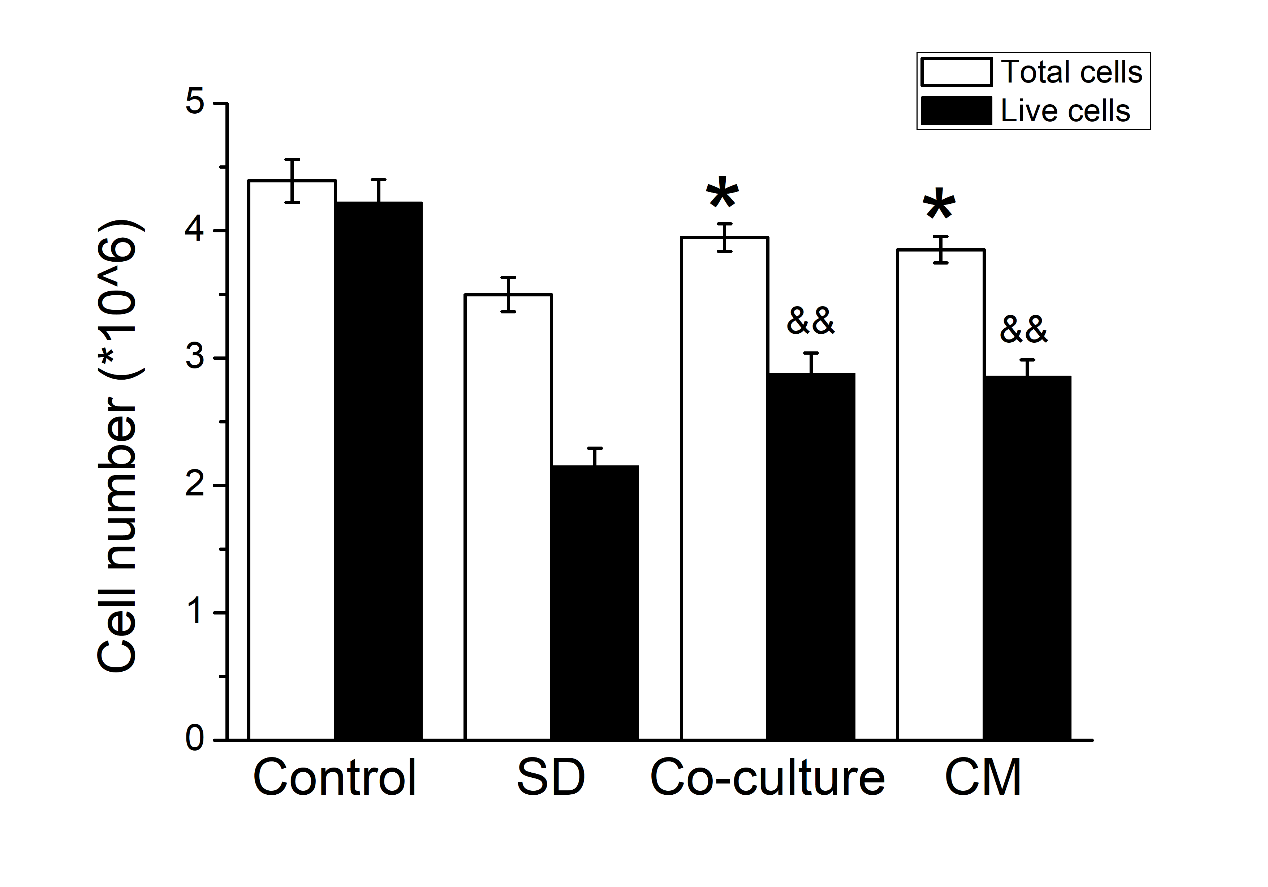
**

**SUPPLEMENTARY FIGURE 2**
